# Comparative Analysis of Repeatability in CT Radiomics and Dosiomics Features under Image Perturbation: A Study in Cervical Cancer Patients

**DOI:** 10.3390/cancers16162872

**Published:** 2024-08-18

**Authors:** Zongrui Ma, Jiang Zhang, Xi Liu, Xinzhi Teng, Yu-Hua Huang, Xile Zhang, Jun Li, Yuxi Pan, Jiachen Sun, Yanjing Dong, Tian Li, Lawrence Wing Chi Chan, Amy Tien Yee Chang, Steven Wai Kwan Siu, Andy Lai-Yin Cheung, Ruijie Yang, Jing Cai

**Affiliations:** 1 Department of Health Technology and Informatics, The Hong Kong Polytechnic University, Hong Kong, China; 21039045r@connect.polyu.hk (Z.M.); jiang.zhang@connect.polyu.hk (J.Z.); yanjing.dong@connect.polyu.hk (Y.D.);; 2 Department of Radiation Oncology, Cancer Center, Peking University Third Hospital, Beijing 100191, China; lx1634@buaa.edu.cn (X.L.);; 3 School of Physics, Beihang University, Beijing 102206, China; 4 Comprehensive Oncology Centre, Hong Kong Sanatorium & Hospital, Hong Kong, China; 5 Department of Clinical Oncology, Queen Mary Hospital, Hong Kong, China; 6 Department of Clinical Oncology, St. Paul’s Hospital, Hong Kong, China

**Keywords:** radiomics, repeatability, dosiomics, cervical cancer, image perturbation, quantitative imaging analysis

## Abstract

**Simple Summary:**

This study aims to evaluate the repeatability of radiomics and dosiomics features via image perturbation of patients with cervical cancer. After analyzing the CT image and dose maps of the 304 included cervical cancer patients, we found higher repeatability of radiomics features than dosiomics features in general. More repeatable features were observed when extracted from the original, Large-sigma Laplacian-of-Gaussian (LoG) filtered, and LLL-/LLH-wavelet filtered images. In addition, a positive correlation was found between image entropy and high-repeatable feature number, regardless of data modality. These findings highlight the potential of radiomics features for robust quantitative imaging analysis in cervical cancer patients, while suggesting the need for further refinement of dosiomics approaches to enhance their repeatability.

**Abstract:**

This study aims to evaluate the repeatability of radiomics and dosiomics features via image perturbation of patients with cervical cancer. A total of 304 cervical cancer patients with planning CT images and dose maps were retrospectively included. Random translation, rotation, and contour randomization were applied to CT images and dose maps before radiomics feature extraction. The repeatability of radiomics and dosiomics features was assessed using intra-class correlation of coefficient (ICC). Pearson correlation coefficient (r) was adopted to quantify the correlation between the image characteristics and feature repeatability. In general, the repeatability of dosiomics features was lower compared with CT radiomics features, especially after small-sigma Laplacian-of-Gaussian (LoG) and wavelet filtering. More repeatable features (ICC > 0.9) were observed when extracted from the original, Large-sigma LoG filtered, and LLL-/LLH-wavelet filtered images. Positive correlations were found between image entropy and high-repeatable feature number in both CT and dose (r = 0.56, 0.68). Radiomics features showed higher repeatability compared to dosiomics features. These findings highlight the potential of radiomics features for robust quantitative imaging analysis in cervical cancer patients, while suggesting the need for further refinement of dosiomics approaches to enhance their repeatability.

## 1. Introduction

Cervical cancer poses a significant healthcare burden, underscoring the necessity for precise and individualized treatment approaches [1]. Texture analysis technology allows for the evaluation of spatial and statistical voxel intensity distributions within an image, thereby providing valuable information about patterns and voxel correlations [2]. In the context of cervical cancer, texture analysis has demonstrated promising potential in improving diagnostic precision, predicting treatment response, and facilitating personalized treatment planning [1,3,4,5].

Radiomics has emerged as a critical component in tailoring personalized treatment strategies and monitoring treatment response by analyzing an extensive array of quantitative features extracted from medical images [6,7,8,9,10]. Chiappa et al. extracted radiomics features from the preoperative ultrasound images and developed machine learning models to accurately predict the malignancies of ovarian masses in the AROMA pilot study [11]. Promising performance of radiomics diagnosis was also reported by the same group in classifying the malignancies of uterine mesenchymal lesions [12]. Radiomics has also demonstrated the potential in treatment response prediction, such as the neoadjuvant chemotherapy response for patients with cervical cancer [13]. Dosiomics, an emerging field built upon principles developed in radiomics, focuses on extracting high-dimensional data from three-dimensional radiation dose distributions to aid in clinical decision-making [14]. The integration of dosiomics in radiation therapy has received significant attention due to its potential applications in modeling normal tissue complications, predicting radiation-induced toxicity, and forecasting tumor control outcomes [15,16]. Dosiomics entails the quantitative evaluation of dosimetric parameters, including maximum dose, mean dose, and dose homogeneity, to comprehensively assess the spatial and dosimetric attributes of the tumor and surrounding tissues during radiation therapy. These features offer insights into the intricate details of dose distribution within the treatment region. Simultaneously, radiomics delves into the complexities of medical images, extracting texture, shape, and intensity features that provide valuable information about tumor heterogeneity, microenvironment, and underlying molecular characteristics [17,18].

Notwithstanding the potential advantages of dosiomic and radiomic features in the management of cervical cancer, it is imperative to conduct a comprehensive investigation into their stability. Variations in imaging acquisition protocols, encompassing variances in scanners, imaging parameters, and segmentation methodologies, have the potential to introduce inherent variability into the extracted features [19]. Furthermore, the presence of image noise, stemming from factors such as suboptimal image quality or motion artifacts, can significantly influence the stability and reproducibility of dosiomic and radiomic features [20]. Gaining a comprehensive understanding of the repeatability of dosiomics and radiomics features is crucial for their successful integration into routine clinical practice [21]. This knowledge ensures the reliability and consistency of feature extraction, enabling accurate and robust analysis for tasks such as treatment response prediction, treatment planning optimization, and patient stratification [22,23]. Ultimately, such insights have profound implications for improving treatment outcomes and optimizing personalized care for cervical cancer patients. While existing research has predominantly focused on the repeatability of radiomics features derived from CT and MR images using test-retest imaging and multiple delineations, limited attention has been given to studying dosiomics features due to the challenges associated with obtaining repeated measurements [24]. Furthermore, inconsistent repeatability results have been observed across different image modalities and cancer sites, thus limiting the generalizability of these findings to new radiomics studies [25,26].

In this study, we aimed to investigate the repeatability of dosiomics and radiomics features extracted from planning CT and dose maps of patients with cervical cancer. We first assessed the repeatability of radiomics and dosiomics features using image perturbations and contour randomizations on different organs at risk (OARs). They were compared both continuously and in binary forms as repeatable and non-repeatable features, followed by analyzing their associations with image/dose appearance. Our findings will provide the first reference of dosiomics feature repeatability for cervical cancer and reveal the confounding factors for radiomics feature reproducibility. This will guide the reproducible dosiomics feature selection for further research endeavors and help to reach a consensus on radiomics feature repeatability under different scenarios.

## 2. Materials and Methods

### 2.1. Patient Dataset

We retrospectively recruited cervical cancer patients with age >18 years old and received complete curative radiotherapy courses from 2012 to 2022. Patients with missing treatment planning CT and dose data and metal artifact in the planning CT except from intrauterine contraceptive device were excluded. A total of 304 patients were included in this study, and the planning CT images and dose maps were collected from the treatment planning system in DICOM format. All CT scans were conducted using a Brilliance Big Bore CT scanner (Philips Healthcare, Amsterdam, The Netherlands). The scanning parameters included a tube voltage of 120 kV, an exposure of 300/325 mAs, an image resolution of 512 × 512, a pixel size of 0.98 × 0.98 mm^2^, and a slice thickness of 5 mm. Five distinct Regions of Interest (ROIs) were delineated manually by radiation oncologists with over 5 years of experience. These ROIs comprised the clinical tumor volume (CTV), Bladder, Rectum, Left Femoral (LFemoral), and Right Femoral (RFemoral). The contouring of the CTV adhered to the updated RTOG protocol released in 2021 [27].

### 2.2. Image Perturbations

**Figure 1:**
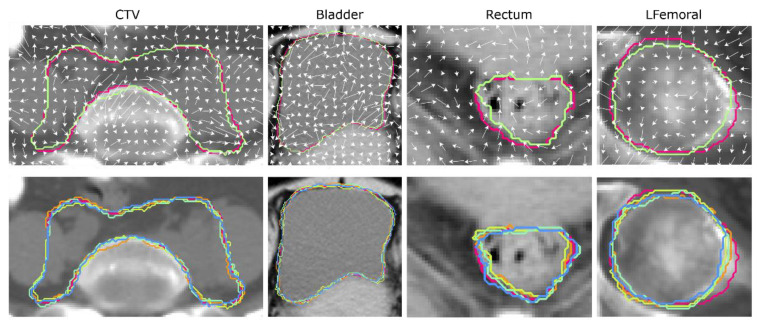
The first row shows random displacement fields (white arrows) in CTV, bladder, rectum, and LFemoral and two randomized contours for the four ROIs (red: original contour, light green: randomized contour) overlayed with the CT image. The second row shows four different randomized contours with different colored lines in addition to the original contour (red line).

Image perturbations were performed by random translations and rotations accompanied by contour randomizations. Each image was translated by 0, 0.4, or 0.8 pixel and rotated by −20, 0, or 20 degrees 40 times. The translation and rotation parameters were chosen randomly for each perturbation. Contour randomizations simulate multiple delineations of the same structure. A 3D random displacement field deforms the segmented mask and results in a randomized contour. The approach for generating random displacement fields is derived from the methodology introduced by Simard et al. [28]. In this adaptation, random vector components for the x and y dimensions were randomly generated following a uniform distribution ranging from −1 to 1 for each voxel point. Notably, no deformations were introduced along the *z*-axis due to the slice-by-slice contouring procedure typically employed in clinical settings. Subsequently, these field vectors were normalized across all three dimensions using the root mean square method. To ensure a smooth and continuous transition in the random displacement fields and prevent abrupt changes in the deformed contours, a Gaussian filter with a sigma value of 5 was applied. Figure 1 shows one example of random displacement field for the studied ROIs (except RFemoral since it is highly symmetrical to LFemoral), and the original and the corresponding randomized contour are visualized by the red and green lines, respectively. Four randomized contours in different colors and the original contour in red are superimposed in the second row of Figure 1. An in-house developed Python program was used to perform image perturbation. The code and examples can be found on our GitHub page (https://github.com/John136219655/Cervical-Cancer-Radiomics-and-Dosiomics-Repeatability-Comparison, accessed on 16 August 2024).

### 2.3. Radiomics Feature Extraction

**Figure 2:**
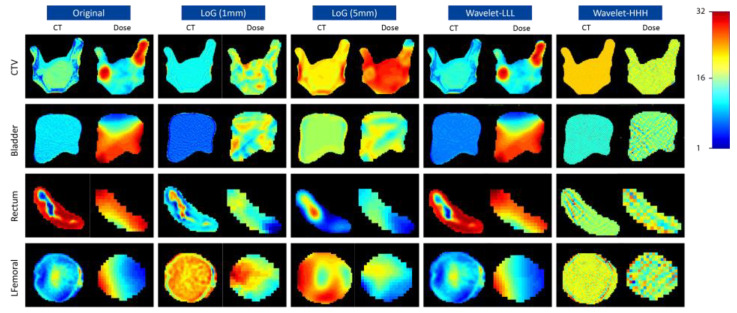
The original, Laplacian-of-Gaussian (LoG) filtered (sigma = 1 and 5 mm), and wavelet (LLL, HHH) filtered images of CT and dose maps within CTV, bladder, rectum, and LFemoral of one example patient. All the images were preprocessed by a 32-bin-number gray level discretization with the final pixel values ranging from 0 to 31. A jet colormap was used to present the voxel values with blue colors for smaller values and red colors for larger values.

The same set of radiomics features were extracted from the CT and dose maps for the five ROIs on each pair of the perturbed image and segmentation. Features extracted from the dose maps were considered as dosiomics in this study. The extracted features encompassed a comprehensive set, including 18 first-order statistics, 24 gray level co-occurrence matrix (GLCM) features, 14 gray level dependency matrix (GLDM) features, 16 gray level run length matrix (GLRLM) features, 16 gray level size zone matrix (GLSZM) features, and 5 neighboring gray-tone difference matrix (NGTDM) features. Each CT image/dose map was filtered using three-dimensional Laplacian-of-Gaussian (LoG) filters with five different sigma values (1, 2, 3, 4, and 5 mm), as well as the complete set of eight coif1 wavelet filters (different combinations of high- and low-pass on each dimension) [29]. All the original and filtered images were further discretized by a fixed bin number of 32. One example of preprocessed CT and dose images under different filters is shown in Figure 2. In total, 1302 features were extracted for each CT/dose map, ROI, and perturbation. The open-source Python package PyRadiomics (version 3.0.0) was used to perform radiomics feature extraction.

### 2.4. Feature Repeatability Analysis

The one-way, random, absolute intra-class correlation coefficient (ICC) was used to assess the repeatability of each radiomics/dosiomics feature against the 40 perturbations. It was calculated according to the following equation:

(1) $$\frac{MS_{R}-MS_{W}}{MS_{R}+\left(k+1\right)MS_{W}}$$

where $MS_{R}$ represents the mean square error of feature values across patients, $MS_{W}$ is the mean square errors across perturbations averaged along patient, and k is the number of perturbations. The binarized feature repeatability was measured using the ICC threshold of 0.9, where a feature was considered high-repeatable if ICC ≥ 0.9 and low-repeatable if ICC < 0.9. The threshold was determined based on previous publications [30] on radiomics feature repeatability analysis.

### 2.5. Statistical Analyses

We compared the radiomics and dosiomics repeatability in both continuous and binarized forms. The average ICC value for each image filter and feature class was first calculated and compared. Comparisons on binarized feature repeatability were presented by highlighting the ratios of commonly high-/low-repeatable features and disagreements between CT and dose on different image filters and feature classes. In order to explain the similarities and differences among different image filters and between the two data modalities, we analyzed the correlations between inherent image characteristics and feature repeatability. The mean values of the entropy, uniformity, and variance of the preprocessed image among all the patients were calculated for each image filter and ROI. These three metrics measure the complexity, heterogeneity, and contrast level, respectively, and were directly acquired from the original first-order radiomics features. Their definitions can be found in the PyRadiomics documentation. The Pearson correlation coefficient (r) was then used to quantify the correlations between the image characteristic evaluations and the average ICC values of the radiomics features [30]. All the feature repeatability and statistical analyses were carried out in the Python platform, and implementation details can be found on our GitHub page (https://github.com/John136219655/Cervical-Cancer-Radiomics-and-Dosiomics-Repeatability-Comparison, accessed on 16 August 2024).

## 3. Results

**Figure 3:**
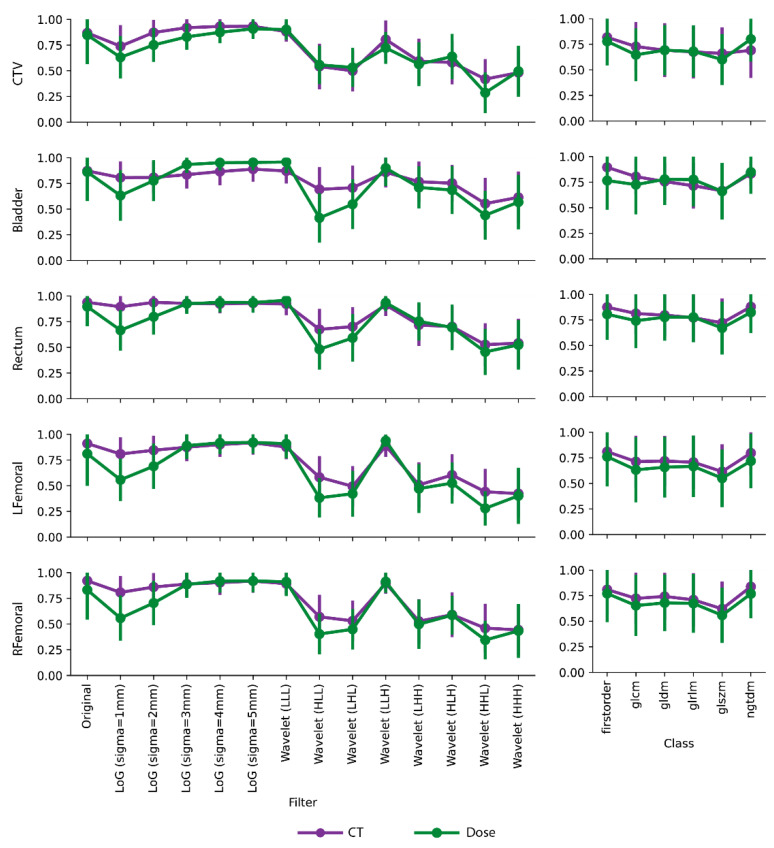
Continuous intraclass correlation coefficient (ICC) comparisons between CT radiomics and dosiomics features on CTV, bladder, rectum, LFemoral, and RFemoral. The mean ICC values averaged on each image filter (left column) and feature class (right column) were plotted as purple (CT) and green (dose) dots with bars indicating the standard deviation. In general, higher mean ICCs were achieved by the CT radiomics compared to dosiomics. Original, large sigma LoG filters, and low-pass wavelet filters resulted in higher mean ICCs compared to other image filters. Rather consistent ICCs were found for different feature classes.

In general, we have observed higher ICC values of radiomics and dosiomics features extracted from the original, large-sigma LoG filtered, and LLL-/LLH-wavelet filtered images. The rest of the wavelet filters yielded significantly lower feature repeatability with average ICC < 0.75, as shown in Figure 3. Fluctuations of mean ICC values were also observed across different feature classes. Specifically, the first-order features exhibited the highest repeatability while the GLSZM features had the lowest. Compared with CT radiomics features, dosiomics feature repeatability were lower, especially after small-sigma LoG and wavelet filtering, and experienced larger deviations across different image filters. One exception on the bladder is that dosiomics features had higher mean ICCs under large-sigma (≥3) LoG filtering. On the contrary, Figure 3 illustrates that feature class had a minimum impact on the consistencies between CT radiomics and dosiomics feature repeatability in terms of mean ICC values.

**Figure 4:**
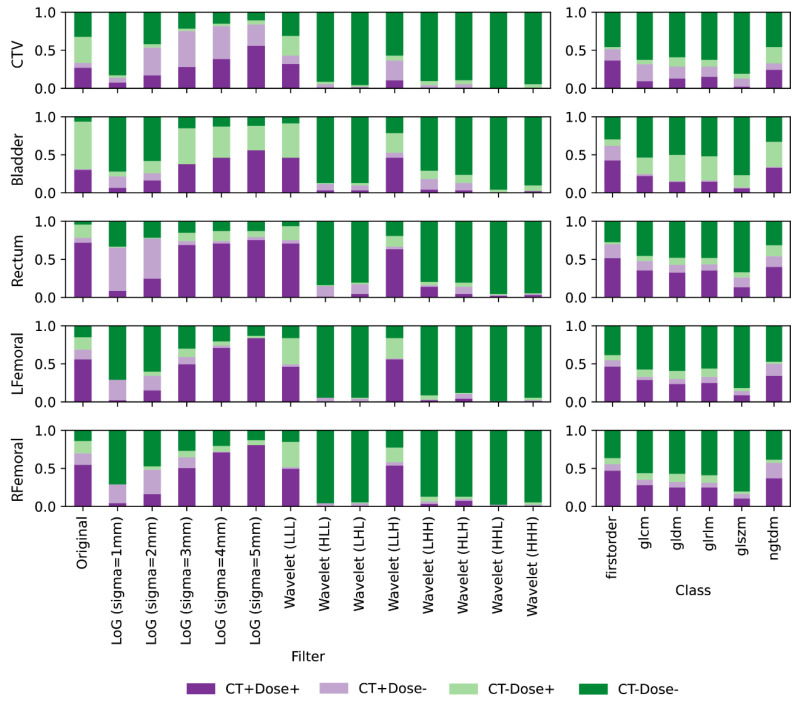
Comparisons of radiomic feature repeatability between CT and planning dose, binarized by the ICC threshold of 0.9. High consistencies can be mostly observed on rectum, LFemoral, and RFemoral for RFs extracted from the original, large sigma (≥3) LoG filtered, and wavelet filtered images/dose maps. Different feature classes demonstrated high consistencies regardless of the ROIs analyzed.

Similar trends can be observed after binarizing the ICC values by the threshold of 0.9, as visualized in Figure 4. More repeatable features were found on the original, large-sigma LoG filtered, and LLL-/LLH-wavelet filtered images. Increasing the sigma values of the LoG filter resulted in more repeatable features. On the other hand, minimum repeatable features were found on the rest of the wavelet filtered images. For feature classes, the first-order class had the largest number of repeatable features while the GLSZM features had the smallest. When comparing the binary consistencies of repeatability between CT radiomics and dosiomics features, large deviations (light green/purple bars) can be observed mostly on CTV, bladder, and rectum. Different image filters also affected the consistency patterns. For example, features that are repeatable in dose but non-repeatable in CT (light green) were mostly found in the original image of the three ROIs, which is different from the mean ICC results. Features that are only repeatable in CT (light purple) were more prevalent in CTV under large-sigma LoG filtering and Rectum under small-sigma LoG filtering.

**Figure 5:**
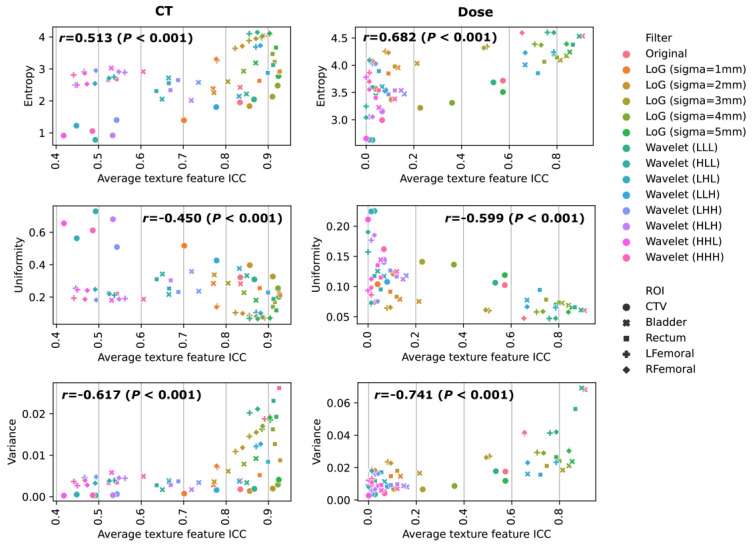
Correlations of mean entropy, uniformity, and variance of preprocessed images with average ICC values of radiomics features at different image filters and ROIs. The Pearson correlation coefficient r and its *p*-value were given on each plot.

Strong correlations between entropy, uniformity, and variance between the preprocessed images and feature repeatability were discovered, regardless of the data modality (Figure 5). The mean entropy, which measures the randomness of the images, had positive correlations with feature repeatability on both CT (r = 0.513) and dose (r = 0.682). A high positive correlation of mean variance (r = 0.617, 0.741) was also observed on CT and dose. On the other hand, the uniformity, which measures the image homogeneity, had a negative correlation (r = −0.450, −0.599).

## 4. Discussion

This study, for the first time, assessed and compared the repeatability of radiomics and dosiomics features from the planning CT and dose maps of primary cervical cancer patients using image perturbation. Features extracted from five different ROIs, including CTV, bladder, rectum, LFemoral, and RFemoral, were independently analyzed. A new contour randomization method was introduced to mimic the manual contouring variations by random deformations. In general, features from large-sigma LoG filtered and LLL-/LLH-wavelet filtered images had higher repeatability, both by absolute and binarized ICC. CT radiomics features presented smaller ICC fluctuations across image filters and had higher repeatability compared to dosiomics, especially on small-sigma LoG filtered and wavelet filtered images. Features from different ROIs also had distinctive repeatability patterns. Further analysis discovered that feature repeatability was highly associated with the randomness, heterogeneity, and contrast of the images, regardless of the data modality. Our findings provided a direct reference of repeatability for both CT radiomics and dosiomics for cervical cancer. The distinctive repeatability patterns for features from different filtered images and different ROIs provided valuable guidance for repeatable feature selection, emphasizing the importance of careful consideration when choosing image filters and defining ROIs. The comparison between CT radiomics and dosiomics shed light on the performance and repeatability of features from different image modalities. It further contributed to the understanding of the strengths and limitations of radiomics and dosiomics, which enables researchers to leverage the rich textural information and clinical relevance of CT radiomics while harnessing the quantitative dose assessment and potential for personalized treatment planning offered by dosiomics, ultimately impacting the development of personalized treatment strategies and improving the reliability and clinical relevance of cancer research.

Our quantitative analysis on the direct impact of image characteristics on feature repeatability may help to explain the different repeatability patterns observed in this study. As suggested in Figure 5, features from images with a higher entropy, lower uniformity, and higher variance are less susceptible to image perturbations. After gray-level discretization with fixed bin counts, images with a higher pixel complexity, heterogeneity, and contrast levels tend to have reduced noise, which can be directly observed from the example images in Figure 2. Therefore, the local pixel connectivity was enhanced, and the resulting texture matrices were more robust against translation and rotation randomizations. Such correlation also raises the importance of image preprocessing where, for example, image resegmentation, image thresholding, and gray-level discretization settings could greatly impact the pixel heterogeneity and noise levels, and careful consideration should be made to balance the repeatability and sensitivity of the extracted features.

For features from LoG filtered images, a higher repeatability was observed as sigma values increased. This is evidenced by the higher mean ICC values and larger repeatable feature numbers with ICC > 0.9. The LoG filter is commonly used for enhancing the visibility of edges and texture in the image. As indicated by Figure 2 and Figure 5, larger sigma values resulted in smoother edge enhancements, which increased the pixel complexities, heterogeneities, and contrast levels and eventually improved consistencies in the extracted features. The impact of wavelet filters on feature repeatability is an important consideration in our study. Wavelet filters decompose an image into high/low frequency bands, enabling the identification and study of fine-scale details as well as coarse-scale structures. In our study, we found that specific combinations of wavelet coefficients, such as LLL, and LLH, exhibited the highest repeatability compared to other combinations. This indicates that these coefficients effectively captured the relevant structural and textural information while minimizing noise and artifacts. However, it is noteworthy that the use of high pass filters in the x and y directions resulted in relatively lower feature repeatability; the high pass filters tend to enhance noise and fine-scale features, making the extracted features more susceptible to variability and less consistent across different images. Moreover, it is important to highlight the high repeatability observed even when applying high-pass filtering on the z-direction. This can be attributed to the rotation along the axial direction, which results in minimal pixel variations along the z-direction.

For radiomics features from CT, the higher repeatability observed in the rectum, RFemoral, and LFemoral ROIs compared to bladder and CTV could stem from the inherent anatomical and tissue characteristics of these regions. The rectum and femoral regions had more complex tissue compositions with high electron density differences, as shown in the example images in Figure 2. Such tissue characteristics were consistent with the quantitative image descriptions in Figure 5 where higher entropy, lower uniformity, and higher variance were exhibited, which led to more repeatable radiomics features. On the other hand, the bladder and CTV regions may have greater complexities in shape but rather similar tissue compositions, leading to increased contour variabilities but decreased pixel complexities and subsequently lower repeatability in the extracted features. For radiomics features from dose data, the higher feature repeatability observed in the rectum and bladder ROIs can be attributed to the nature of dosimetry data. Dosiomics involves the analysis of radiation dose distribution within the target area and surrounding organs. The rectum and bladder, being adjacent to the target area, experienced a sharper dose drop-off and higher dose variance within the ROI volume, resulting in the dosiomics features being less susceptible to perturbations.

Several limitations should be acknowledged in this study. Firstly, the relatively small sample size may restrict the generalizability of the findings and limit the statistical power to detect subtle differences. The single-center design introduces the possibility of bias related to patient selection, imaging protocols, and data acquisition techniques, which may affect the external validity of the results. Additionally, the absence of external validation using an independent dataset limits the ability to confirm the repeatability findings in different settings or populations. The focus on specific data modalities, such as CT scans and dose maps, overlooks the potential variability and repeatability of features derived from other imaging modalities, such as MRI or PET. Moreover, the study did not directly assess the clinical impact of feature repeatability on treatment outcomes or patient management decisions. Future research should address these limitations by incorporating larger and more diverse cohorts, conducting multi-center studies with standardized protocols, performing external validation, exploring feature repeatability across various imaging modalities, and investigating the clinical implications of feature repeatability in real-world scenarios.

## 5. Conclusions

In conclusion, this study investigated the repeatability of CT radiomics and dosiomics features under image perturbations. The findings suggest that CT-based radiomics features exhibit higher repeatability compared to features derived from dose maps. The higher repeatability of CT-based radiomics features highlights their potential as reliable and consistent quantitative markers in imaging-based analyses. Our findings contribute to the development of more reliable imaging biomarkers for personalized cancer treatment planning and response assessment. Further research is needed to explore the impact of feature repeatability on predictive performance and clinical utility under different settings and patient populations.

## Data Availability

The feature tables and statistical analysis results presented in this study are available in this article. Further inquiries on the raw image and segmentation data can be directed to the corresponding author. https://github.com/John136219655/Cervical-Cancer-Radiomics-and-Dosiomics-Repeatability-Comparison (accessed on 16 August 2024).
